# Insights into Enhanced Capacitive Behavior of Carbon Cathode for Lithium Ion Capacitors: The Coupling of Pore Size and Graphitization Engineering

**DOI:** 10.1007/s40820-020-00458-6

**Published:** 2020-06-06

**Authors:** Kangyu Zou, Peng Cai, Baowei Wang, Cheng Liu, Jiayang Li, Tianyun Qiu, Guoqiang Zou, Hongshuai Hou, Xiaobo Ji

**Affiliations:** 1 College of Chemistry and Chemical Engineering, Central South University, Changsha, 410083 People’s Republic of China; 2 College of Metallurgy and Chemical Engineering, Jiangxi University of Science and Technology, 86 Hongqi Road, Ganzhou, 341000 People’s Republic of China

**Keywords:** Carbon materials, Pore size regulation, Graphitization, Capacitive behavior, Lithium ion capacitor

## Abstract

The lack of methods to modulate intrinsic textures of carbon cathode has seriously hindered the revelation of in-depth relationship between inherent natures and capacitive behaviors, limiting the advancement of lithium ion capacitors (LICs). Here, an orientated-designed pore size distribution (range from 0.5 to 200 nm) and graphitization engineering strategy of carbon materials through regulating molar ratios of Zn/Co ions has been proposed, which provides an effective platform to deeply evaluate the capacitive behaviors of carbon cathode. Significantly, after the systematical analysis cooperating with experimental result and density functional theory calculation, it is uncovered that the size of solvated PF_6_^−^ ion is about 1.5 nm. Moreover, the capacitive behaviors of carbon cathode could be enhanced attributed to the controlled pore size of 1.5–3 nm. Triggered with synergistic effect of graphitization and appropriate pore size distribution, optimized carbon cathode (Zn_90_Co_10_-APC) displays excellent capacitive performances with a reversible specific capacity of ~ 50 mAh g^−1^ at a current density of 5 A g^−1^. Furthermore, the assembly pre-lithiated graphite (PLG)//Zn_90_Co_10_-APC LIC could deliver a large energy density of 108 Wh kg^−1^ and a high power density of 150,000 W kg^−1^ as well as excellent long-term ability with 10,000 cycles. This elaborate work might shed light on the intensive understanding of the improved capacitive behavior in LiPF_6_ electrolyte and provide a feasible principle for elaborate fabrication of carbon cathodes for LIC systems.

**Electronic supplementary material:**

The online version of this article (10.1007/s40820-020-00458-6) contains supplementary material, which is available to authorized users.

## Highlights

- Pore size and graphitization engineering of carbon cathode were orientated-designed by regulating the molar ratios of Zn/Co ions.
- Zn_90_Co_10_-APC and its assembled PLG//Zn_90_Co_10_-APC LIC both deliver the excellent electrochemical performances.

**Electronic supplementary material:**

The online version of this article (10.1007/s40820-020-00458-6) contains supplementary material, which is available to authorized users.

## Graphical abstract

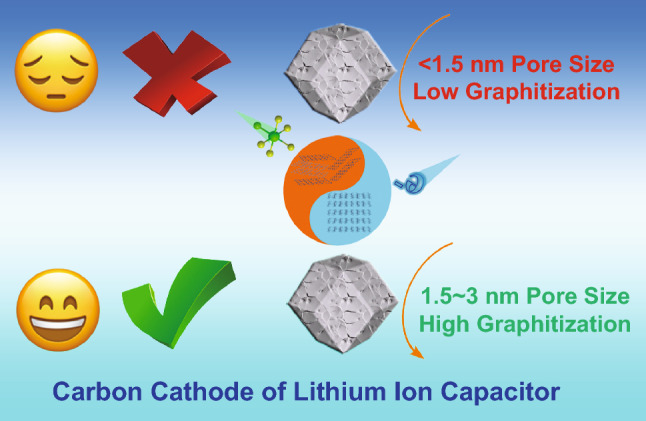

## Introduction

Lithium ion capacitor (LIC) is assembled by a battery-type anode and a capacitor-type cathode in a Li-salt electrolyte, elaborately combining the merits of high energy/power densities and long cycle life [1–4]. Nevertheless, the current electrochemcial performances of LICs are unsatisfactory, which are limited by the kinetic discrepancy between the sluggish intercalation/deintercalation mechanism of anode and fast adsorption/desorption behavior of cathode [5–7]. At the present time, enormous endeavors have been put into the researches for improving electrochemical kinetics of lithium intercalated/deintercalated behavior of carbon anodes [8–10]. It must be noticeable that energy/power densities of LICs could be influenced by the electrochemical performances of cathode, due to the equation of 1/*C*_cell_ = 1/*C*_anode_ + 1/*C*_cathode_ [11]. Therefore, the fabrication of high-performance carbon cathode is the crucial step for development of LICs. However, limited by uncontrolled intrinsic textures, the desired carbon cathodes could be hardly engineered. For example, however the widely used activated carbon (AC) cathodes (e.g., YP-50) have relatively large specific surface area (~ 1500 m^2^ g^−1^), delivering unfavorable specific capacity (< 40 mAh g^−1^). The presence of this dissatisfactory circumstance is ascribed to the narrow microporosity of AC, which brings about the inefficiently storage of solvated PF_6_^−^ ions [12]. Moreover, the low specific capacity exacerbates the difficulty of electrode fabrication, seriously hindering the improvement of LICs [13, 14]. To tackle this encountered problem, surface functionalization process has been implemented, which effectively improves the specific capacitance of commercial AC powder [15]. Furthermore, involving the pseudocapacitance reaction to carbon electrodes by heteroatom-doping method is a feasible strategy for promoting fast reaction kinetics and providing the additional capacity [16–18]. However, these aspects are not considered as the crucial factors for determining the intrinsic electrochemical performances of electric double-layer capacitors (EDLCs) [19].

It is well known that the energy storage mechanism of EDLCs is governed by ion electrosorption at the electrode/electrolyte surface and the electrochemical performances of EDLCs are mainly depended on the porous characteristics [20]. Thus, an appropriate pore size has been identified as a crucial parameter for boosting the electrochemical performance of EDLCs [21]. Generally, when the pore size of carbon matches with the solvated ion size, the maximized capacitance could be obtained. As a result, the optimization of pore size for carbon has been accounted as the key point to enhance the capacitance of EDLCs [22–24]. In addition, the graphitization also plays a vital role for increasing the capacitance of carbon materials, resulted from a high degree of electronic conductivity, which is difficultly adjusted [25]. Thus, a convenient and effective strategy for directionally adjusting pore size and graphitization of carbon materials should be urgently explored, which is beneficial to provide a fundamental outstanding in capacitive behavior. Nowadays, extensive research efforts have been dedicated to optimize the energy density of supercapacitor in tetraethylammonium tetrafluoroborate and ionic liquid electrolytes [26, 27]. Nevertheless, the capacitive behaviors of carbon materials in LiPF_6_ electrolyte have been rarely studied, limiting the progress of LICs. Hence, it is necessary to comprehensively explore the in-depth correlation between the micro-structure characteristics (including pore size dispersion and graphitization) and capacitive behavior of the carbon cathode, which could provide guidelines about improvement of LICs.

Zeolite imidazole frameworks (ZIFs), a well-known subfamily of metal–organic frameworks (MOFs), are constructed from the well-defined metal ions/clusters and organic ligands [28–31]. Due to the intrinsic merits of the highly ordered and adjustable structure, ZIFs have been chosen as an ideal sacrificial precursor for purposefully fabricating the carbon materials with diversified peculiarities [32–35]. In view of the above-mentioned considerations, aiming at the optimization of natures of carbon cathodes, MOF-derived carbon materials with different pore size distribution and graphitization have been orientated-engineered by regulating the molar ratios of Zn/Co ions. After the systematical analysis through the collaborating with experimental result and DFT calculation, attributed to the presence of suitable pore size (1.5–3 nm), the capacitive behaviors of carbon materials could be enhanced on account of the strong adsorption/desorption of solvated PF_6_^−^ ions. And the rate ability of carbon cathode could be boosted by improving the graphitization degree accompanied with enhanced electronic conductivity. Moreover, the improved graphitization and meso/macroporosities are beneficial for boosting surface-induced capacitive behavior of carbon cathode. Significantly, the engineered Zn_90_Co_10_-APC possesses the prominent natures combing the desired graphitization degree and appropriate pore size distribution, delivering superior electrochemical performances. This work provides a feasible strategy to regulate the micro-structure of carbon materials and benefits the deep understanding of the capacitive behaviors of carbon cathode affected by the pore size as well as graphitization, greatly promoting the electrochemical performance of LICs.

## Experimental Section

### Materials and Methods

#### Preparation of Zn_*x*_Co_100−*x*_–ZIFs

For the synthesis of mono-metal-ZIFs (ZIF-8 (Zn_100-_ZIF) or ZIF-67 (Co_100-_ZIF)): Zn(NO_3_)_2_·6H_2_O (3 mmol, 891 mg) or Co(NO_3_)_2_·6H_2_O (3 mmol, 873 mg) was dissolved in 30 mL methanol, respectively. Then, 2-methylimidazole (984 mg, 12 mmol) was dissolved in another 30 mL methanol. Then, the later solution was added to the former solution and stirred. After a few minutes, the consequent solution was kept for 24 h. Finally, the obtained product was washed with methanol and then dried at 80 °C under vacuum.

For the synthesis of bimetal-ZIFs: The preparation process was similar as method above but by regulating the molar ratio of Zn(NO_3_)_2_·6H_2_O and Co(NO_3_)_2_·6H_2_O. Thus, Zn_75_Co_25_-ZIF, Zn_50_Co_50_-ZIF and Zn_25_Co_75_-ZIF were obtained with the different Zn/Co radios (3:1, 1:1, and 1:3), respectively.

Note x represents the proportion of Zn element in Zn_*x*_Co_100−*x*_–ZIFs.

#### Preparation of Zn_*x*_Co_100−*x*_–PCs

Zn_*x*_Co_100−*x*_–ZIFs were heat-treated in a tube furnace under an argon flow at 800 °C for 2 h with a heating rate of 10 °C min^−1^ and then cooled down to room temperature. In order to remove the residual metal impurities, the obtained products were washed with thoroughly HF solution and distilled water. The sample was finally dried under vacuum at 100 °C for 24 h, which was correspondingly labeled as Zn_*x*_Co_100−*x*_–porous carbons (Zn_*x*_Co_100−*x*_–PCs), respectively.

#### Fabrication of Zn_*x*_Co_100−*x*_–APCs

As-prepared Zn_*x*_Co_100−*x*_–PCs were thoroughly mixed with KOH in a mass ratio of 1:1. The mixture was further annealed at 800 °C for 1 h under argon atmosphere with a heating rate of 10 °C min^−1^. The calcined samples were washed with diluted HCl and distilled water. The obtained specimen was dried under vacuum at 100 °C for 24 h, which were correspondingly labeled as Zn_*x*_Co_100−*x*_–APCs, respectively.

### Materials Characterization

The structural information of the obtained samples was characterized by using a powder X-ray diffractometer (XRD, Rigaku) equipped with a Cu-Kα radiation of 0.15418 nm, and Raman spectra were recorded by using Raman spectrometer (DXR, Thermo-Fisher Scientific). N_2_ absorption/desorption isotherms were collected by Micromeritics ASAP 2020 instrument, and the pore size distributions of samples were calculated by non-local density functional theory (NLDFT) method. Moreover, an X-ray photoelectron spectroscopy (XPS, VG Multi Lab 2000 system) was carried out for analyzing the compositions of the products. The surface morphology and inner structure were detected by scanning electron microscopy (SEM, Hitachi S-4800) and high-angle annular dark field scanning transmission electron microscopy (HAADF-STEM, Titan G2 60-300). Electronic conductivity is reciprocal of electronic resistivity, and electronic resistivity of carbon membrane was carried out at room temperature under 0.55 T magnetic field using the van der Pauw method by the Hall measurement (ECOPIA HMS 3000). Moreover, the carbon membrane was prepared with grinding apparatus by using the powder compressing machine under the 10 MPa.

### Electrochemical Measurements

The MOF-derived carbon cathodes were prepared by mixing 80 wt% active materials (Zn_*x*_Co_100−*x*_–PCs and Zn_*x*_Co_100−*x*_–APCs), 10 wt% binder polyvinylidene fluoride (PVDF), and 10 wt% conductive carbon (Super P) in N-methyl pyrrolidinone (NMP). Afterward, the resulting mixtures were coated on an aluminum foil and the as-obtained electrodes were dried at 80 °C for 12 h in a vacuum box. Meanwhile, the commercial graphite anode was acquired by the similar method as the cathode. The mixed slurries were consisted of the 70 wt% active material, 15 wt% binder carboxymethyl cellulose, and 15 wt% conductive carbon (Super P) in deionized water, which were subsequently bushed on a copper foil.

The half-cells of working electrodes (both the anode and cathode) were assembled into a series of CR2016-type coins in the Braun glovebox with high-purity argon atmosphere. The lithium metal was used as the counter and reference electrode, and a Whatman GF/C glass fiber membrane was utilized the separator. Moreover, 1 mol L^−1^ LiPF_6_ solution in ethylene carbonate (EC) and dimethyl carbonate (DMC) (1:1, v/v) with 5 wt% fluoroethylene carbonate was severed as the electrolyte. The graphite anode was cycled for 5 cycles in a half-cell versus Li/Li^+^ under 0.1 A g^−1^, which is beneficial for assembling the LICs.

All electrochemical measurements were measured at room temperature. Cyclic voltammetry (CV) curves with various scan rates and electrochemical impedance spectra (EIS) were measured by a MULTI AUTOLAB M204 (MAC90086). Galvanostatic charge/discharge (GCD) surveys were recorded on an Arbin BT2000 instrument at diverse current densities within an appropriate voltage window. Cycle-life tests for half-cells and LICs were recorded on a battery system (Land CT2001A model).

The specific capacitance (C, F g^−1^), energy density (E, W h kg^−1^), and power density (P, W kg^−1^) of LICs, based on the GCD measurements, can be calculated according to Eqs. 1–3:

1 $$ C = It/\Delta Vm $$

2 $$ E = C(V_{\hbox{max} }^{2} - V_{\hbox{min} }^{2} ) \, /2 \times 3.6 $$

3 $$ P = E \times 3600/t $$

where *V*_*max*_ (V) and *V*_*min*_ (V) are the maximum and minimum discharge potentials, *ΔV* (V) is the potential change, *I* (A) is the discharge current, *t* (s) is the discharge time, and *m* (g) is the total mass of active material in both anode and cathode.

### DFT Calculations

The DFT calculations were calculated for the solvated structures of PF_6_^−^ ion by EC and DMC molecules, utilizing the Perdew–Burke–Ernzerhof (PBE) exchange–correlation functional within the framework of generalized gradient approximation (GGA). The numerical foundational settings were employed for valid computation of wave functions. Moreover, the analytical foundational set functions spend longer than numerical foundational settings. Furthermore, the architectonic optimization of the solvation structures and their energies were obtained by the triple-numerical polarization (TNP) foundational settings (one atomic orbital (AO) for each occupied atomic orbital, the second and the third settings of valence AO’s, *d*-functions for non-hydrogen atoms, and *p*-functions on hydrogen atoms). The combination of the functional and the foundational settings as GGA-PBE/TNP was referred in this system. The calculation for the PF_6_^−^ ion solvation was achieved in the gas phase without the presence of the counter cation Li^+^. All atomic positions were refined during the optimization procedure.

## Results and Discussions

### Characterization and Morphology

**Fig. 1:**
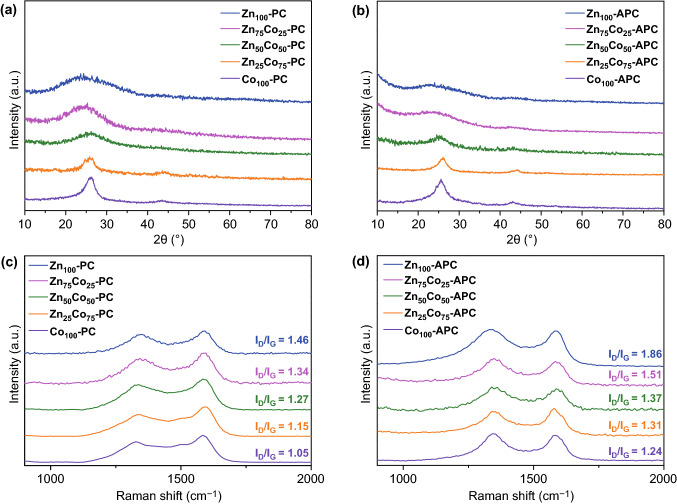
The XRD patterns and Raman spectra of **a**, **b** Zn_*x*_Co_100−*x*_PCs and **c**, **d** Zn_*x*_Co_100−*x*_APCs

It is well known that ZIF-8 and ZIF-67 are isologues, which are fabricated by the reactions of 2-methyl imidazole with zinc and cobalt ions, respectively [36]. Moreover, the bimetallic ZIFs obtained by controlling the molar ratios of zinc to cobalt ions (Zn^2+^/Co^2+^) have been deduced to possess the same crystalline state as monometallic ZIF [37–39]. The as-prepared Zn_*x*_Co_100−*x*_–ZIFs have been firstly characterized by XRD measurements. As shown in Fig. S1, the samples exhibit the same XRD peaks, which certifies that the high phase purity and same topological structure. The experimental XRD pictures of Zn_*x*_Co_100−*x*_–ZIFs are almost the same, which are identical to the corresponding computer-simulated pattern, indicating the isomorphism of Zn_*x*_Co_100−*x*_–ZIFs. As shown in Fig. 1a, two obvious peaks are centered at ~ 25° and ~ 44° which are ascribed to the (002) and (100) planes of carbon, respectively [40, 41]. Due to the broad diffraction peaks with low densities, Zn_100_-PC manifests mainly amorphous characteristic in nature [42–44]. On the other aspect, the full-width at half maximum of the (002) peak of Co_100_-PC becomes narrow, attributed to remarkable graphitization effect of cobalt species triggered by the partially filled 3d orbitals [45–47]. Furthermore, as the proportion of cobalt ions increases, the crystallinities of Zn_*x*_Co_100−*x*_–PCs are gradually improved. Significantly, it is noted that Zn_*x*_Co_100−*x*_–APCs also show the high graphitization degrees even after KOH chemical activation (Fig. 1b). To further verify the graphitization degree of carbon materials, the Raman spectra of Zn_*x*_Co_100−*x*_–PCs and Zn_*x*_Co_100−*x*_–APCs are recorded. As shown in Fig. 1c, d, two characteristic peaks are centered at around 1350 and 1585 cm^−1^, respectively, corresponding to D bands (disorder-induced carbon) and G-bands (graphitized carbon) [48–50]. The relative *I*_*D*_/*I*_*G*_ ratios are 1.46, 1.34, 1.27, 1.15, and 1.05 for Zn_100_-PC, Zn_75_Co_25_-PC, Zn_50_Co_50_-PC, Zn_25_Co_75_-PC, and Co_100_-PC, demonstrating that the graphitization degree of Zn_*x*_Co_100−*x*_–PCs could be enhanced as the proportion of cobalt ions. Meanwhile, the *I*_*D*_/*I*_*G*_ values for the Zn_*x*_Co_100−*x*_–APCs are 1.86 (Zn_100_-APC), 1.51 (Zn_75_Co_25_-APC), 1.37 (Zn_50_Co_50_-APC), 1.31 (Zn_25_Co_75_-APC), and 1.24 (Co_100_-APC), respectively. Moreover, the electronic conductivities of Zn_100_-PC, Zn_75_Co_25_-PC, Zn_50_Co_50_-PC, Zn_25_Co_75_-PC, and Co_100_-PC samples are about 0.10 S cm^−1^, 1.12 S cm^−1^, 5.42 S cm^−1^, 8.70 S cm^−1^, and 12.5 S cm^−1^. And, the electronic conductivities of Zn_100_-APC, Zn_75_Co_25_-APC, Zn_50_Co_50_-APC, Zn_25_Co_75_-APC, and Co_100_-APC samples are approximately 0.05, 0.89, 4.20, 6.70, and 9.50 S cm^−1^, respectively. The results above clearly demonstrate the enhancement of the electronic conductivity stems from the gradually increscent graphitization degree. Therefore, the graphitization degree of carbon materials could be adjusted by regulating the molar ratio of Zn/Co ions.

**Fig. 2:**
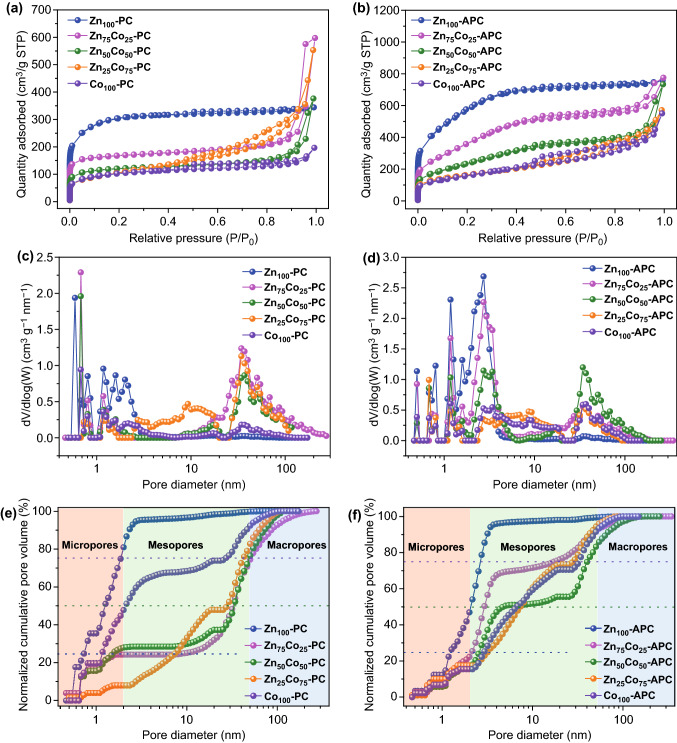
a , **b** N_2_ adsorption–desorption isotherms, **c**, **d** pore size distribution curves and **e**, **f** normalized cumulative pore size distributions of Zn_*x*_Co_100−*x*_–PCs and Zn_*x*_Co_100−*x*_–APCs

The porous features of Zn_*x*_Co_100−*x*_–PCs and Zn_*x*_Co_100−*x*_–APCs have been explored by N_2_ adsorption–desorption isothermal analyses at 77 K, which are summarized in Table 1. As displayed in Fig. 2a, it is obvious that the curve of Zn_100_-PC presents the classical type I, which illustrates the dominant micropore structure, resulted from the leave of Zn vapor at relatively high temperature [51, 52]. It is noteworthy that the typical type H3 hysteresis loop within the *P*/*P*_0_ range of 0.45-1.0 becomes gradually wider as the increased proportion of cobalt source, revealing the existence of meso-/macro-pores with a broad size distribution [53]. This phenomenon mainly is attributed to removal of Co species on the carbon substance. It is found that the BET specific surface areas are gradually decreased in order of Zn_100_-PC (957 m^2^ g^−1^) > Zn_75_Co_25_-PC (524 m^2^ g^−1^) > Zn_50_Co_50_-PC (370 m^2^ g^−1^) > Zn_25_Co_75_-PC (367 m^2^ g^−1^) > Co_100_-PC (332 m^2^ g^−1^). The results above show that carbon structure with different surface area and pore size can be designed by adjusting the molar ratio of Zn/Co ions. It is all known that KOH chemical activation could enlarge the BET specific surface area and change the porosity characteristic of carbon material [54–57]. Thus, it is obvious that Zn_*x*_Co_100−*x*_–APCs exhibit the hierarchical porous structures with enlarged specific surface areas and abundant meso-/macro-porosities (Fig. 2b). In addition, Fig. 2c, d shows the pore size distributions of all samples. In order to further assess the difference in pore size distribution, the normalized cumulative pore size distributions of Zn_*x*_Co_100−*x*_–PCs and Zn_*x*_Co_100−*x*_–APCs have been carefully analyzed (Fig. 2e, f). And, the comparison of normalized cumulative pore volume could offer a direct evidence for assessing the change of pore features [58]. Generally, often-used volume weighted average pore size *d*_50_ does not fully represent the pore size distribution width. Thus, *d*_25_ and *d*_75_, serving as the pore width encompassing 25% and 75% of the total pore volume, are added for showing the comprehensive distribution width of carbon materials, respectively. Significantly, the comprehensive pore distribution is anticipated to provide an important parameter to analyze the relationship between pore size distribution and capacitive behavior of carbon cathode in the later content.

**Table 1:** Summary of the porosity parameters for as-prepared samples

Sample	*S*_BET_	*V*_total_	*V*_mirco_	*d*_25_	*d*_50_	*d*_75_
	(m^2^ g^−1^)	(cm^3^ g^−1^)	(cm^3^ g^−1^)	(nm)	(nm)	(nm)
Zn_100_-PC	957	0.53	0.26	0.73	1.25	1.90
Zn_75_Co_25_-PC	524	0.87	0.19	10.06	33.75	51.60
Zn_50_Co_50_-PC	370	0.58	0.12	1.51	33.40	48.16
Zn_25_Co_75_-PC	367	0.85	0.01	7.52	28.66	42.51
Co_100_-PC	332	0.29	0.06	1.18	2.07	27.27
Zn_100_-APC	2073	1.17	0.30	1.23	2.12	2.64
Zn_75_Co_25_-APC	1385	1.16	0.23	2.11	2.96	17.37
Zn_50_Co_50_-APC	881	1.09	0.42	2.51	4.68	40.07
Zn_25_Co_75_-APC	585	0.88	0.03	3.19	7.31	28.61
Co_100_-APC	573	0.84	0.04	2.93	6.71	33.08
Zn_90_Co_10_-APC	1535	1.18	0.24	1.81	2.82	7.17

**Fig. 3:**
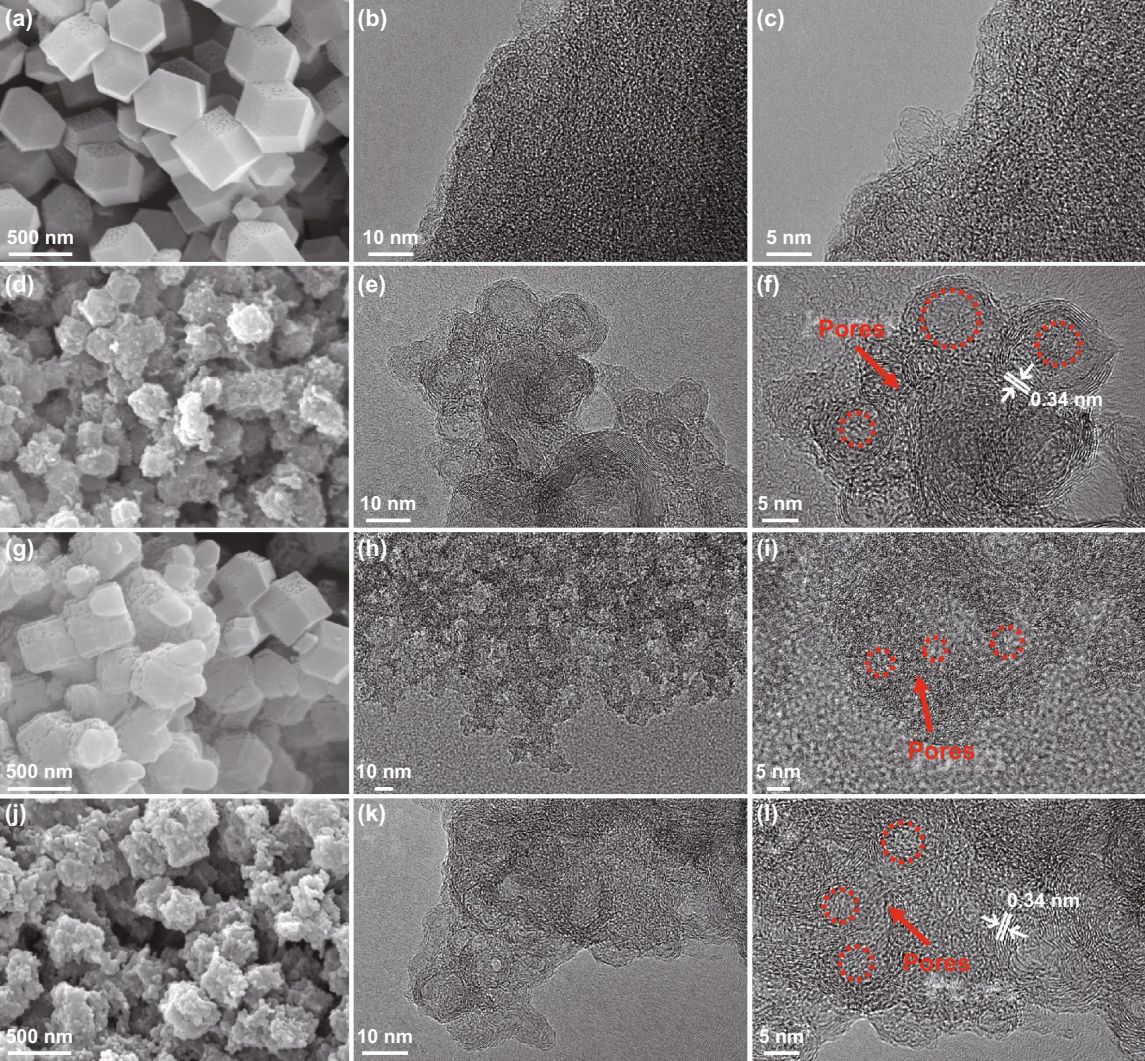
SEM and HRTEM images of **a-c** Zn_100_-PC, **d-f** Co_100_-PC, **g-i** Zn_100_-APC and **j-l** Co_100_-APC

In order to intuitively observe the changes of morphologies and porous structures, the SEM and HRTEM images of Zn_100_-PC, Co_100_-PC, Zn_100_-APC, and Co_100_-APC are displayed in Fig. 3. The above samples manifest rhombic dodecahedral shape. Significantly, it is found that Zn_100_-PC distinctly is the amorphous characteristic with dominated microporous feature and Co_100_-PC manifests the graphitized carbon layers with prominent meso-/macro-porosities. Furthermore, the graphitic ribbons with 0.34 nm lattice fringes are noticed [59]. Meanwhile, after KOH chemical activation, Zn_100_-APC obviously possesses the meso-/macro-porosities and Co_100_-APC exhibits the uniform and shrunken meso-/macro-pores. These discussed results are well consistent with the above-mentioned analyzed results of XRD, Raman and porosity.

### Electrochemical Performance of Carbon Cathode

**Fig. 4:**
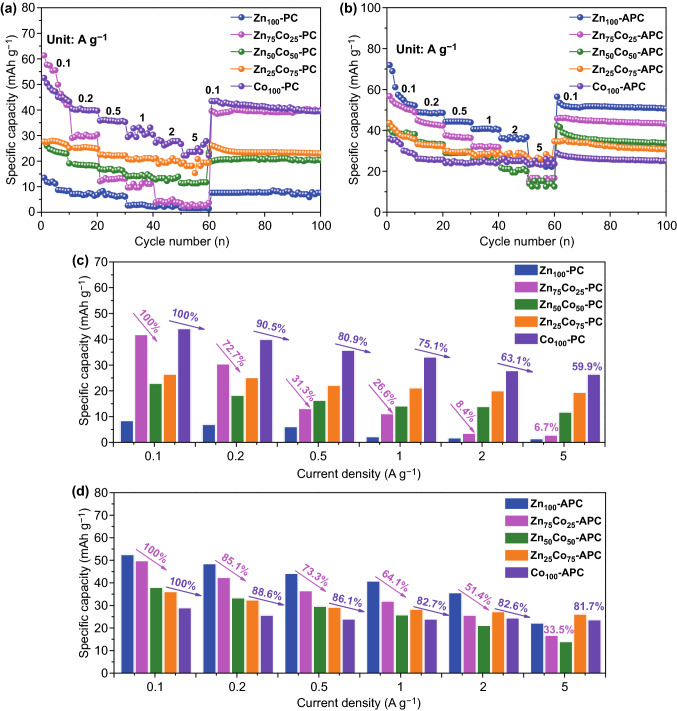
Rate capabilities and comparisons of specific capacities at different current densities of **a**, **c** Zn_*x*_Co_100−*x*_–PCs and **b**, **d** Zn_*x*_Co_100−*x*_–APCs

To evaluate the adsorption/desorption of the solvated anion (PF_6_^−^) behaviors of the obtained specimens as the cathodes of LICs, the half-cell tests versus lithium metal have been investigated within the potential range of 2.0–4.5 V. As shown in Fig. 4a-d, rate performances of Zn_*x*_Co_100−*x*_**–**PCs and Zn_*x*_Co_100−*x*_**–**APCs at various current densities are presented. Moreover, the corresponding coulombic efficiencies have been shown in Figs. S2 and S3. It is noticed that Zn_100_-PC possesses the worst electrochemical performances among the Zn_*x*_Co_100−*x*_**–**PCs, only delivering 15 mAh g^−1^ at 0.1 A g^−1^. Although featured with relatively large BET specific surface area, the electrochemical properties of Zn_100_-PC are seriously restricted by the narrow and inappropriate pore characteristics [60]. Specially, prominent microporous characteristic of Zn_100_-PC (80% microporous volume proportion) has been presented. On the contrary, although possessing the relatively low BET specific surface area, the specific capacity of Co_100_-PC could be reached up to 40 mAh g^−1^, resulted from the abundant meso-/macro-porosities. Combining with Fig. 2e, f, Co_100_-PC possesses suitable pore size range (*d*_50_ = 2.07 nm ~ *d*_75_ = 27.27 nm), resulting in the strong adsorption/desorption behaviors of solvated PF_6_^−^ ion. The results above demonstrate that the adsorption/desorption of the solvated anion (PF_6_^−^) behaviors are mainly determined by the pore sizes of carbon materials and an appropriate pore size is more crucial than a high surface area in order to obtain high values of capacitance. Interestingly, after KOH chemical activation, Zn_100_-APC with enhanced electrochemical performances delivers a reversible specific capacity of 55 mAh g^−1^ at 0.1 A g^−1^, whereas the electrochemical performances of Co_100_-APC are obviously decreased. The results above illustrate that the electrochemical performances of Zn_100_-APC and Co_100_-APC are affected by the changed pore size distributions. It is found that the microporous volume proportion of Zn_100_-APC decreased to about 49%, and *d*_50_ and *d*_75_ of Zn_100_-APC are increased up to 2.12 and 2.64 nm, respectively, revealing the enlarged pore size distribution is beneficial for the adsorption/desorption of solvated PF_6_^−^ ion. In addition, the d_50_ of Co_100_-APC have changed to 6.71 nm and the reduced electrochemical performances of Co_100_-APC are attributed to the less porosity of about 2 nm. Meanwhile, *d*_25_ (10.06 nm), *d*_50_ (33.75 nm), and *d*_75_ (51.60 nm) of Zn_75_Co_25_-PC are altered to 2.11, 2.96, and 17.37 nm after chemical activation. Because of the increased pore volume proportion in 2 ~ 3 nm, the electrochemical performance of Zn_75_Co_25_-APC has been significantly improved. The above-mentioned results demonstrate that 2 ~ 3 nm pore size could bring out the strong adsorption/desorption behavior of solvated PF_6_^−^ ions. Moreover, it is obvious that the rate performances of carbon materials derived from Zn_*x*_Co_100−*x*_**–**ZIFs with relatively high Co content could be boosted, resulting from the graphitization effect accompanied with enhanced electronic conductivity [61]. Furthermore, the XPS measurements have been carried out to verify the effect on capacity of N species. The XPS survey spectra and the elemental contents of Zn_*x*_Co_100−*x*_**–**PCs and Zn_*x*_Co_100−*x*_**–**APCs are presented in Fig. S4 and Table S1, respectively. The results demonstrate that Zn_*x*_Co_100−*x*_**–**APCs almost exhibit the similar chemical environment, which could further highlight the effect of pore sizes of carbon materials for the capacity contribution. In addition, Zn_100_-PC with relatively high N contents of 20.59% possesses the dissatisfactory electrochemical performances, which illustrates that the N-doping effect is not preponderant in the capacity contribution and the appropriate pore size plays the vital role in the high capacity.

**Fig. 5:**
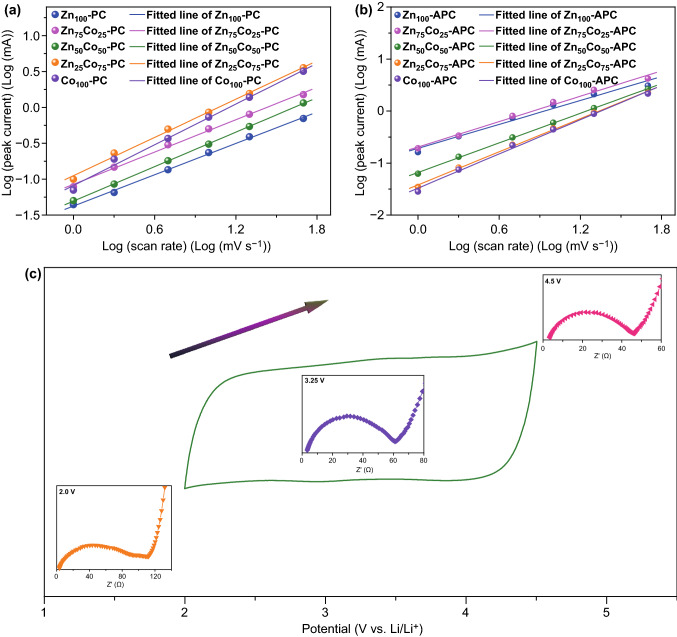
Determination of the *b*-value using the relationship between the peak current and the scan rate of **a** Zn_*x*_Co_100−*x*_–PCs and **b** Zn_*x*_Co_100−*x*_–APCs. **c** Illustration of variation of Nyquist plots under different open-circuit voltages by taking Co_100_-APC as an example

In order to better understand the adsorption/desorption behavior of the obtained material for solvated PF_6_^−^ ion, kinetic analysis based on CV measurements with different scan rates range from 1 to 50 mV s^−1^ is carried out (Figs. S5 and S6). The CV curves of Zn_*x*_Co_100−*x*_**–**PCs and Zn_*x*_Co_100−*x*_**–**APCs at different scan rates show a quasi-rectangular shape with slight deformation, illustrating a dominant electric double-layer behavior with pseudocapacitance contribution. In addition, the CV curves of Zn_*x*_Co_100−*x*_**–**PCs and Zn_*x*_Co_100−*x*_**–**APCs show the unobvious deformation at the high scan rate as the proportion of cobalt ions increases, attributing to the graphitic crystallization with enhanced electron transfer. Generally, two types of charge-storage mechanisms are widely discussed, namely the surface-induced capacitive behavior and the diffusion-controlled process [62, 63]. The surface-induced capacitive behavior includes the faradaic behavior from the surface redox reaction and the electrical double-layer behavior from ion adsorption/desorption. The diffusion-controlled process is primarily originated from insertion of electrolyte ions. The peak current (i) and the scan rate (ν) follow the power law, which are shown in Eqs. 4 and 5 [64–66]:

4 $$ i = a\nu^{b} $$

5 $$ \log \left( i \right) = b\log \left( \nu \right) + \log a $$

where *a* and *b* stand for variable parameters. Importantly, on account of the *b*-value, the different electrochemical storage behavior can be classified. When *b*-value is close to 0.5, it indicates the dominating diffusion-controlled process. On the contrary, when the *b*-value is near 1, the storage capacity mostly originates from the surface-induced capacitive process. Figure 5a, b shows the fitted lines of Zn_*x*_Co_100−*x*_–PCs and Zn_*x*_Co_100−*x*_–APCs derived from the anodic peaks located 3.25 V and the corresponding *b*-values are calculated. The *b*-value of Zn_100_-PC, Zn_75_Co_25_-PC, Zn_50_Co_50_-PC, Zn_25_Co_75_-PC, and Co_100_-PC is 0.73, 0.75, 0.80, 0.88, and 0.94, respectively, revealing that the intensive graphitization could boost the surface-induced capacitive kinetics. Importantly, after KOH chemical activation, the *b*-value of Zn_100_-APC, Zn_75_Co_25_-APC, Zn_50_Co_50_-APC, Zn_25_Co_75_-APC and Co_100_-APC is reached up to 0.76, 0.81, 0.94, 1.06, and 1.09, respectively, demonstrating that the surface-induced capacitive behavior could be improved by increasing meso/macroporosities. The results above illustrate that increased electronic conductivity triggered by graphitization and rapid ion transport originated from meso/macroporosities could synergistically boost the surface-induced capacitive behavior. In other words, surface-induced capacitive behavior of carbon material could be promoted by enhancing the graphitization and meso/macroporosities.

As shown in Figs. S7 and S8, the Nyquist plots consisted of an intercept of the real axis about *R*_s_ (the resistance of materials in the cell, containing electrode, electrolyte, and separation), a semicircle in middle–high-frequency about *R*_ct_ (the resistance of charge transfer) and a sloping straight line in low frequency about *Z*_W_ (corresponding to the capacitive behavior linked with the PF_6_^−^ ion adsorption in the whole porous carbon network) [67–69]. Moreover, it is obvious that the diameter of the semicircle at medium–high-frequency region decreases as there is an increase in potential from 2 to 4.5 V, showing the occurrence of the absorption of PF_6_^−^ anion on the surface of electrode material, which can enhance the electronic conductivity (Fig. 5c). The *σ*_W_ values can be obtained from the slope of the Z′ and ω^−1/2^ plot in the Warburg region, which could estimate the PF_6_^−^ transfer. The enlargement of the graphitization degree with the proportion of cobalt ions could reduce the *σ*_W_ value, attributing to the enhancement of the electronic conductivity. Moreover, after 100 cycles at 0.1 A g^−1^, *R*_ct_ and *σ*_W_ values of Zn_*x*_Co_100−*x*_–PCs and Zn_*x*_Co_100−*x*_–APCs are increased under different open-circuit voltages, which indicates the deteriorated performance, resulted from the destroyed structures of electrode materials.

### Theoretical Calculation and Analysis of Solvated PF_6_^−^ Ion

**Fig. 6:**
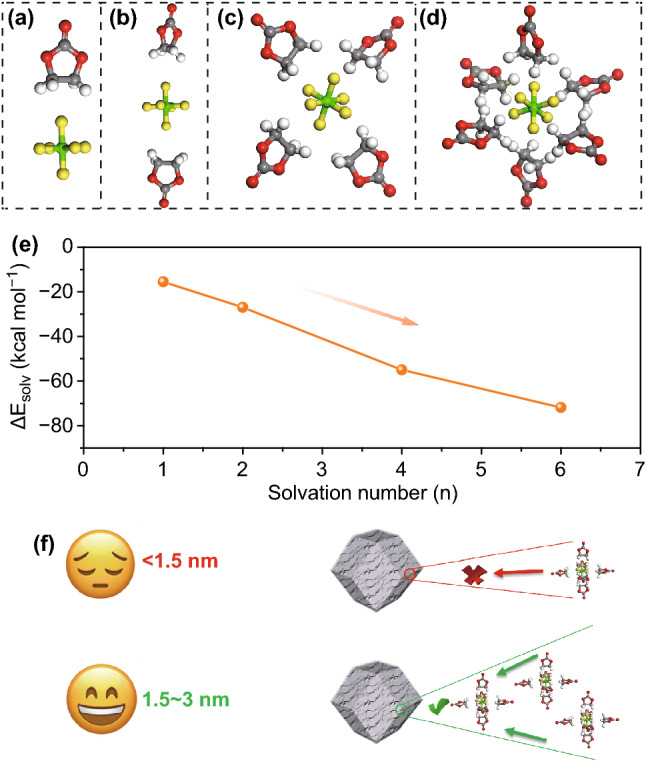
Optimized solvation structures of PF_6_^−^(EC)_*i*_ (*i* = 1, 2, 4, 6) by DFT calculations: **a** PF_6_^−^(EC)_1_, **b** PF_6_^−^(EC)_2_, **c** PF_6_^−^(EC)_4_ and **d** PF_6_^−^(EC)_6_. **e** Variation tendency of solvation energies of PF_6_^−^(EC)_*i*_ as a function of the solvation number. **f** Schematic illustration of electrosorption of solvated PF_6_^−^ ions with a finite pore size

The DFT calculations have been performed to gain insight into the optimal structure of solvated PF_6_^−^ ion. The solvation structures of PF_6_^−^ ion have been firstly examined in EC molecules [70–72]. The optimized structures for PF_6_^−^(EC)*i* (*i *= 1, 2, 4, 6) have been present in Fig. 6a-d, respectively. In view of the asymmetric nature of the EC molecule, the position of the methyl group was switched prior to each optimization until the lowest energy solvation structure was found. Moreover, the solvation energy (Δ*E*_solv_) has been calculated by Eq. 6:

6 $$ \Delta E_{\text{solv}} = E\left[ {PF_{6}^{ - } \left( {EC} \right)_{i} } \right] - E\left[ {PF_{6}^{ - } } \right] - E\left[ {EC} \right] \, x \, i $$

where E[PF_6_^−^(EC)_*i*_] represents the electronic energy for PF_6_^−^(EC)_*i*_, E[PF_6_^−^] represents the electronic energy for the PF_6_^−^ ion, E[EC] represents the electronic energy for the solvent EC molecule, and i is the solvation number. Table S2 lists the specific calculated values of solvation energies of PF_6_^−^(EC)_*i*_ structures, and Fig. 6e shows the variation tendency of solvation energy as a function of the solvation number. Importantly, it is found that the solvation process of PF_6_^−^ ion accompanying with EC is spontaneous and the PF_6_^−^(EC)_6_ possesses the lowest solvation energy of − 71.84 kcal mol^−1^. Meanwhile, the DMC molecule has also been examined for the solvation structures of PF_6_^−^ ion and the related solvation energies of PF_6_^−^(DMC)_*i*_ structures are listed in Fig. S9 and Table S3. Note that the EC molecule is inclined to combine with PF_6_^−^ ion to form the stable solvation structure and the PF_6_^−^(EC)_6_ is the optimized solvation structure. Thus, the optimized solvation structure of PF_6_^−^(EC)_6_ mainly exists in the 1 mol L^−1^ LiPF_6_ solution system (EC and DMC (1:1, v/v) with 5 wt% FEC) and the size of PF_6_^−^(EC)_6_ structure is about 1.5 nm [73]. The DFT calculation greatly supports the experimental results above, which reveals that the pores smaller than solvated PF_6_^−^ ions size of about 1.5 nm are inaccessible for energy storage and the pore size of carbon substantially becomes larger than the size of solvated PF_6_^−^ ions to adequately accommodate diffuse charge layers. Moreover, in consideration of the compact layers from adjacent pore walls, the pores lower than twice the size of the solvated PF_6_^−^ ions could bring out desired capacitive performances (Fig. 6f). In summary, on the basis of DFT calculations, 1.5 ~ 3 nm pore size could trigger strong adsorption/desorption behavior of solvated PF_6_^−^ ions, which is well consistent with experimental results above.

### Electrochemical Performance of LICs

**Fig. 7:**
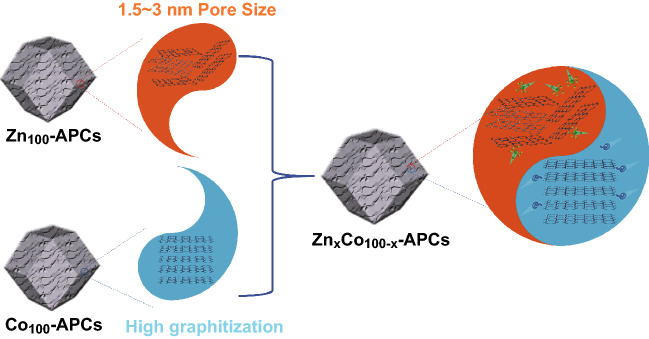
Schematic illustration of the synthesis of the desired carbon cathode through the ingenious incorporation of high graphitization and appropriate pore size distribution

**Fig. 8:**
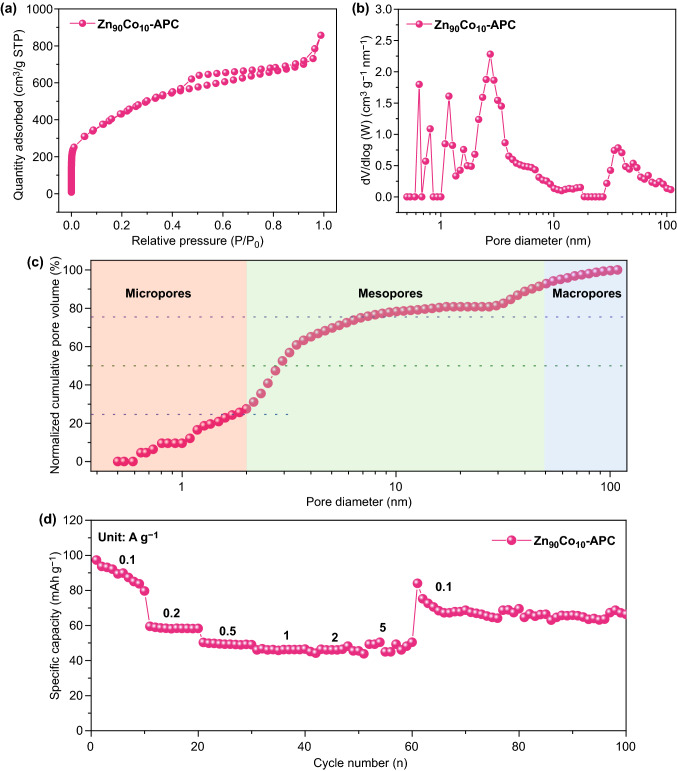
**a** N_2_ adsorption–desorption isotherms, **b** pore size distribution curves, **c** normalized cumulative pore size distributions and **d** rate capabilities of Zn_90_Co_10_ -APC

**Fig. 9:**
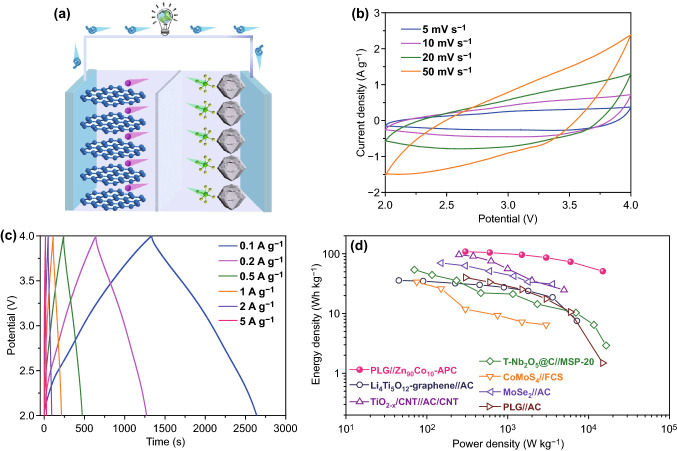
**a** Schematic illustration of the charge-storage mechanisms for the LICs. **b** CV curves and **c** GCD profiles of PLG//Zn_90_Co_10_-APC LIC. **d** Ragone plots of this work compared with other reported literatures

In considerations of the results above, through the ingenious incorporation of high graphitization and appropriate pore size distribution, desired carbon cathode could be reasonably engineered and Zn_90_Co_10_-APC has been elaborately designed (Figs. 7 and S10). Thanks to the synergistic effect of graphitization and appropriate pore size distribution, Zn_90_Co_10-_APC exhibits the most outstanding adsorption/desorption behavior of solvated PF_6_^−^ ions among the as-prepared carbon samples (Fig. 8 and Table 1). Zn_90_Co_10-_APC shows a high specific capacity of ~ 100 mAh g^−1^ at the current density of 0.1 A g^−1^ and could still retain 50 mAh g^−1^ even at the high current density of 5 A g^−1^, showing an excellent rate capability. Generally, commercialize LICs are generally composed of graphite anode and AC cathode. The theoretical capacity of graphite anode is 372 mAh g^−1^, and the detailed electrochemical performances have been recorded (Fig. S11). In order to evaluate the superiority of Zn_90_Co_10-_APC sample as the cathode, the pre-lithiated graphite (PLG) as anode of LIC has been utilized in this work and the related schematic illustration of the charge-storage mechanisms is shown in Fig. 9a. As shown in Fig. 9b, c, the ideal rectangular shape CV curves with slight deviations are observed and the slopes of GCD profiles are not strictly linear with unobvious distortion, demonstrating the collaboration of two types of charge-storage mechanisms (faradaic and non-faradaic behaviors) [74–76]. The specific capacitances (based on the total mass of cathode and anode) of PLG//Zn_90_Co_10_-APC LIC are 65.1, 62.9, 57.6, 51.7, 44.1, and 30.55 F g^−1^ at the current densities of 0.1, 0.2, 0.5, 1, 2, and 5 A g^−1^, respectively. Notably, PLG//Zn_90_Co_10_-APC LIC presents the high energy density of 108 Wh kg^−1^ at power density of 300 W kg^−1^. The energy density could still maintain 51 Wh kg^−1^ at a relatively high power density of 15,000 W kg^−1^. Moreover, as shown in Figs. 9d and S12, the PLG//Zn_90_Co_10_-APC LIC presents superior energy/power characteristics compared to other previously reported LICs, such as Li_4_Ti_5_O_12_-graphene//AC [77], TiO_2-x_/CNT//AC/CNT [78], *T*-Nb_2_O_5_@C//MSP-20 [79], CoMoS_4_//FCS [80], MoSe_2_//AC [81], Fe_3_O_4_@C//a-EW-NaCl [82], and CNF//PANi@CNF-10 [83], and PLG//AC. Significantly, the PLG//Zn_90_Co_10_-APC LIC exhibits the good long-term ability with 10,000 cycles in the potential window of 2.0-4.0 V (Fig. S13). Moreover, in consideration of the potential drop (ΔV) originated from the GCD curves during the cyclic process, the potential drop of PLG//AC LIC is increased up to 1.4 V, which is much larger than that of PLG//Zn_90_Co_10_-APC LIC, further revealing the PLG//Zn_90_Co_10_-APC LIC possesses better long-term ability compared to PLG//AC LIC (Fig. S14). This study is anticipated to offer an in-depth understanding of capacitive behavior of carbon cathode in LiPF_6_ electrolyte and afford more possibilities for directionally fabricating desired carbon cathode of high-performance LICs.

## Conclusion

In this work, an orientated-designed pore size and graphitization engineering strategy of the carbon materials based on ZIFs has been successfully developed, greatly promoting comprehensive exploration of the relationship between the intrinsic features and capacitive behaviors of carbon cathodes. Meaningfully, it is found that an appropriate pore size is more important than a high surface area for enhanced capacity and the suitable pore sizes of 1.5 ~ 3 nm could match well with the solvated PF_6_^−^ ion, bringing out the strong adsorption/desorption behavior. Significantly, in the LiPF_6_ electrolyte, the rate ability could be improved by enhancing the graphitization degree of carbon materials. Furthermore, it is found that the surface-induced capacitive kinetics of carbon could be promoted by enhancing the graphitization and meso/macroporosities. It is found that pore size of 1.5–3 nm could trigger strong adsorption/desorption behavior of solvated PF_6_^−^ ions according to DFT calculations. Notably, thanks to the synergistic effect of graphitization and appropriate pore size distribution, Zn_90_Co_10_-APC shows the most excellent electrochemical performance and the assembly PLG//Zn_90_Co_10_-APC LIC exhibits the superior electrochemical performances compared to PLG//AC LIC. This in-depth investigation based on the fundamental understanding of capacitive behavior in LiPF_6_ electrolyte can offer the directed guidances for the rational design of carbon cathodes for high-performance LICs.

## Electronic supplementary material

Below is the link to the electronic supplementary material.

Supplementary material 1 (PDF 1665 kb)
