# Supplementary material for: Insights into Enhanced Capacitive Behavior of Carbon Cathode for Lithium Ion Capacitors: The Coupling of Pore Size and Graphitization Engineering
Source: Nanomicro Lett. 2020 Jun 6;12:121. doi: 10.1007/s40820-020-00458-6 (PMC7770892; doi:10.1007/s40820-020-00458-6)
Supplement: Supplementary file 1 — Supplementary material 1 (PDF 1665 kb) [file 40820_2020_458_MOESM1_ESM.pdf]

Supporting Information for

Insights into Enhanced Capacitive Behavior of Carbon Cathode for Lithium Ion Capacitors: The Coupling of Pore Size and Graphitization Engineering

Kangyu Zou[1], Peng Cai[1], Baowei Wang[1], Cheng Liu[1], Jiayang Li[1], Tianyun Qiu[1], Guoqiang Zou[1,*], Hongshuai Hou[1], Xiaobo Ji[1,2]

[1]College of Chemistry and Chemical Engineering, Central South University, Changsha 410083, People's Republic of China

[2]College of Metallurgy and Chemical Engineering, Jiangxi University of Science and Technology, 86 Hongqi Road, Ganzhou 341000, People's Republic of China

*Corresponding author. E-mail: gq-zou@csu.edu.cn (Guoqiang Zou)

Supplementary Figures and Tables

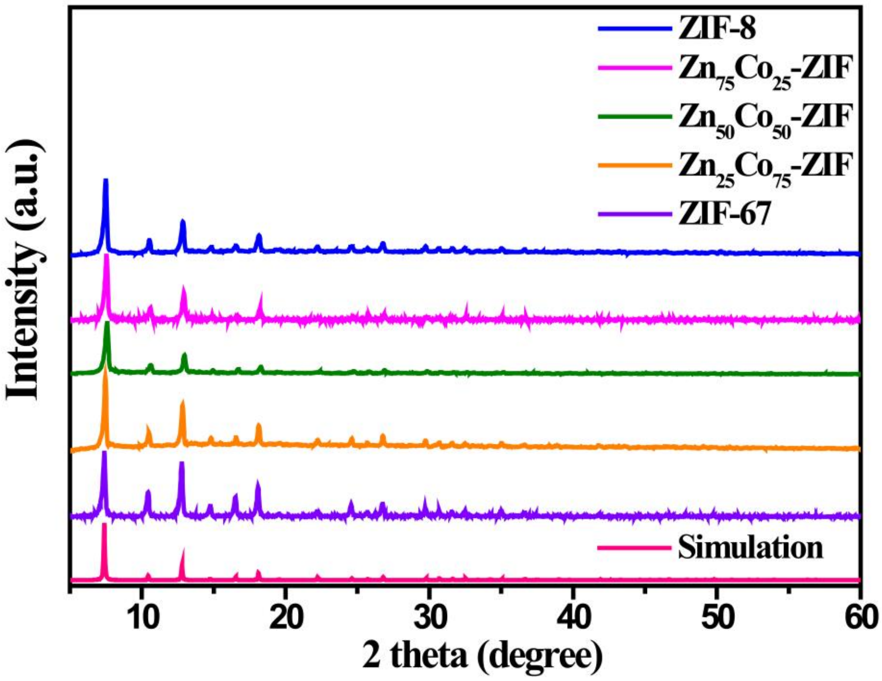

Fig. S1 Simulated and experimental XRD patterns of Zn_xCo_{100-x}-ZIFs

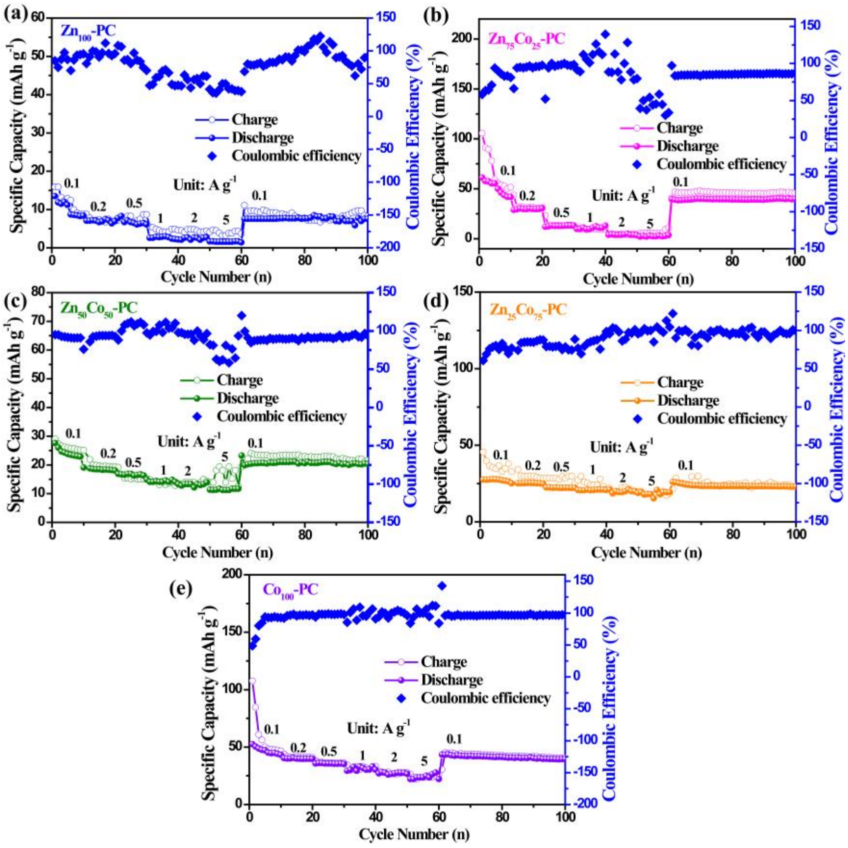

Fig. S2 The coulombic efficiencies of the Zn_xCo_{100-x}-PCs

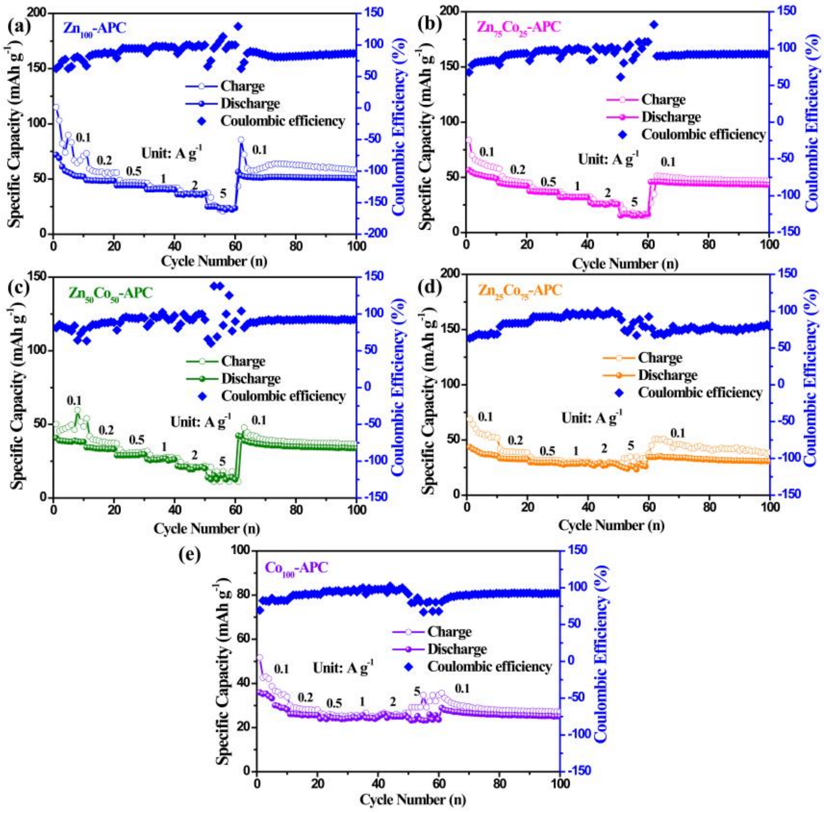

Fig. S3 The coulombic efficiencies of the Zn_xCo_{100-x}-APCs

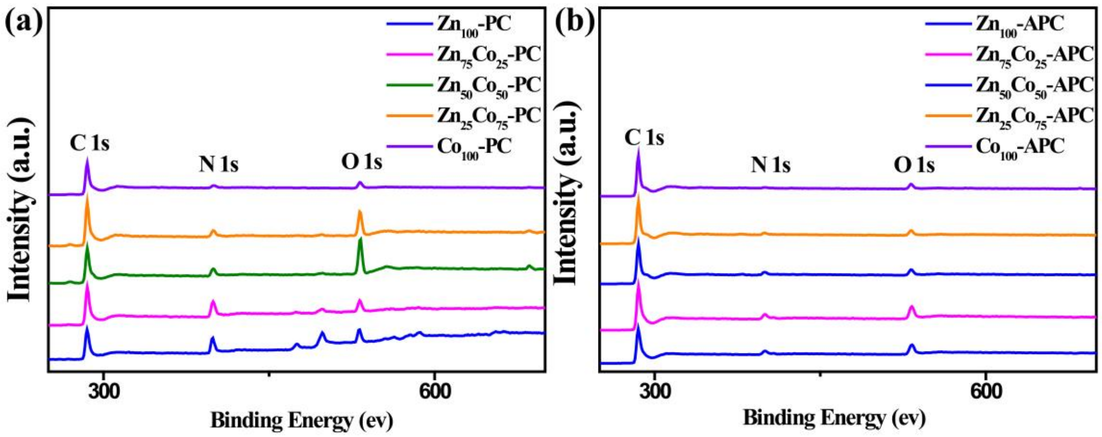

Fig. S4 XPS survey spectra of the of Zn_xCo_{100-x}-PCs and Zn_xCo_{100-x}-APCs

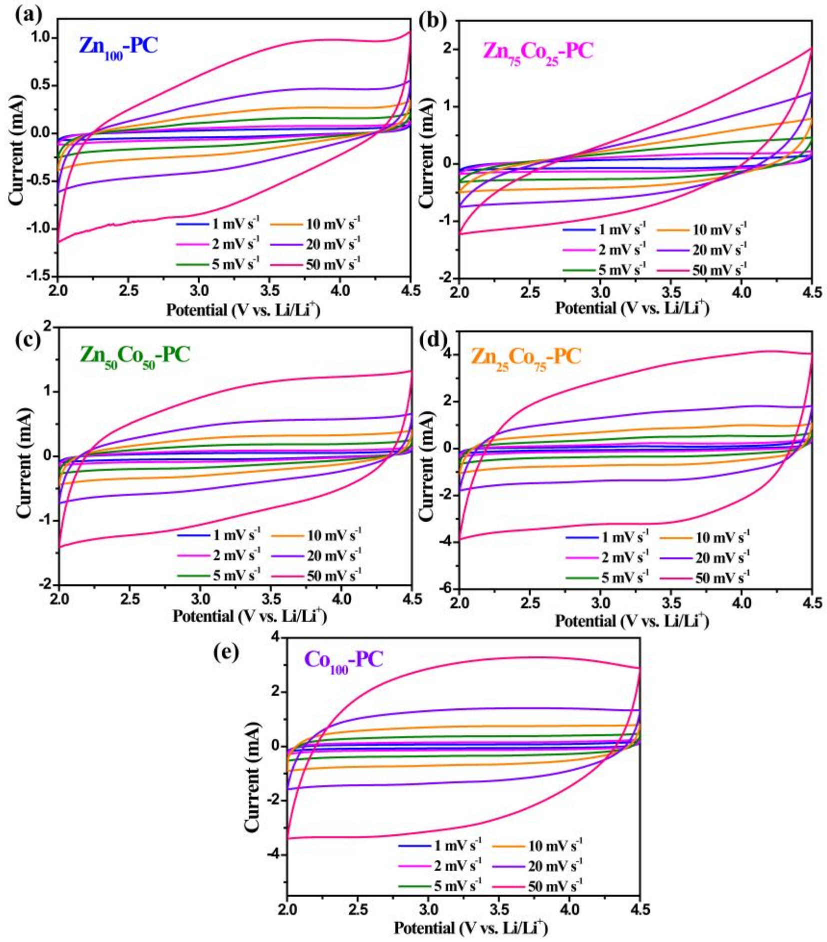

Fig. S5 CV curves of (**a**) Zn_{100}-PC, (**b**) $Zn_{75}Co_{25}$-PC, (**c**) $Zn_{50}Co_{50}$-PC, (**d**) $Zn_{25}Co_{75}$-PC and (**e**) Co_{100}-PC cathodes at various scan rates from 1 to 50 mV s^{-1}

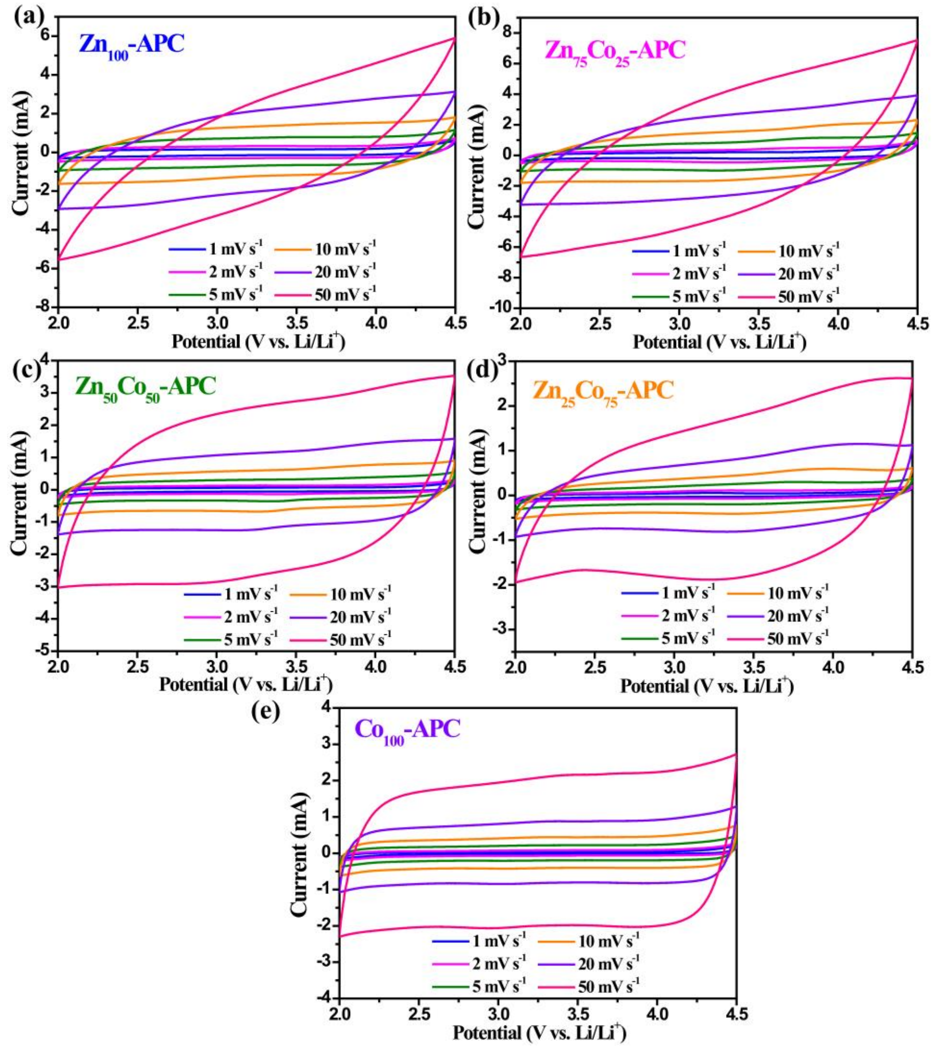

Fig. S6 CV curves of (**a**) Zn_{100}-APC, (**b**) $Zn_{75}Co_{25}$-APC, (**c**) $Zn_{50}Co_{50}$-APC, (**d**) $Zn_{25}Co_{75}$-APC and (**e**) Co_{100}-APC cathodes at various scan rates from 1 to 50 mV s^{-1}

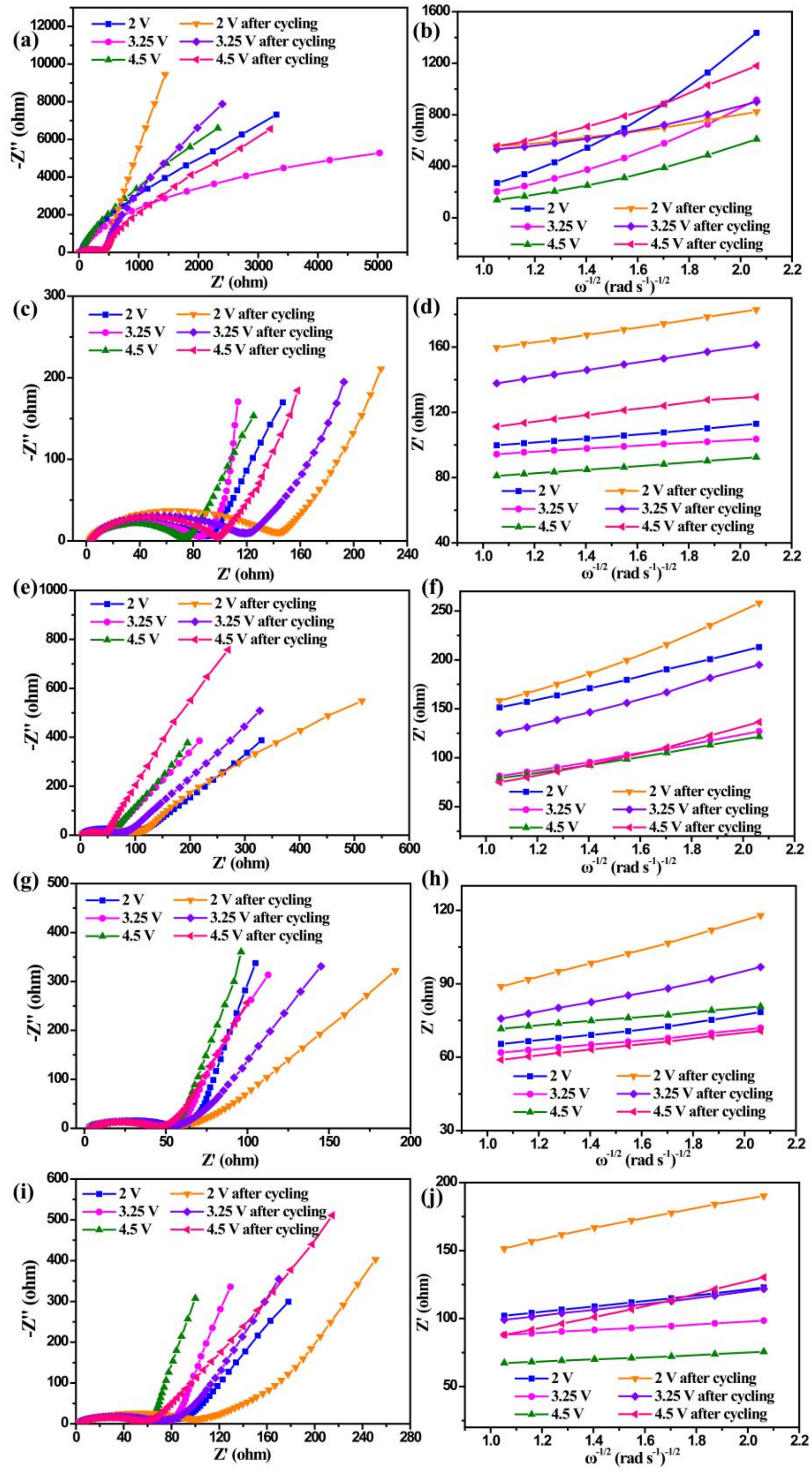

Fig. S7 Nyquist plots and relationships of Z' and $\omega^{-1/2}$ in the low frequency region of (**a**, **b**) Zn_{100}-PC, (**c**, **d**) $Zn_{75}Co_{25}$-PC, (**e**, **f**) $Zn_{50}Co_{50}$-PC, (**g**, **h**) $Zn_{25}Co_{75}$-PC, (**i**, **j**) Co_{100}-PC

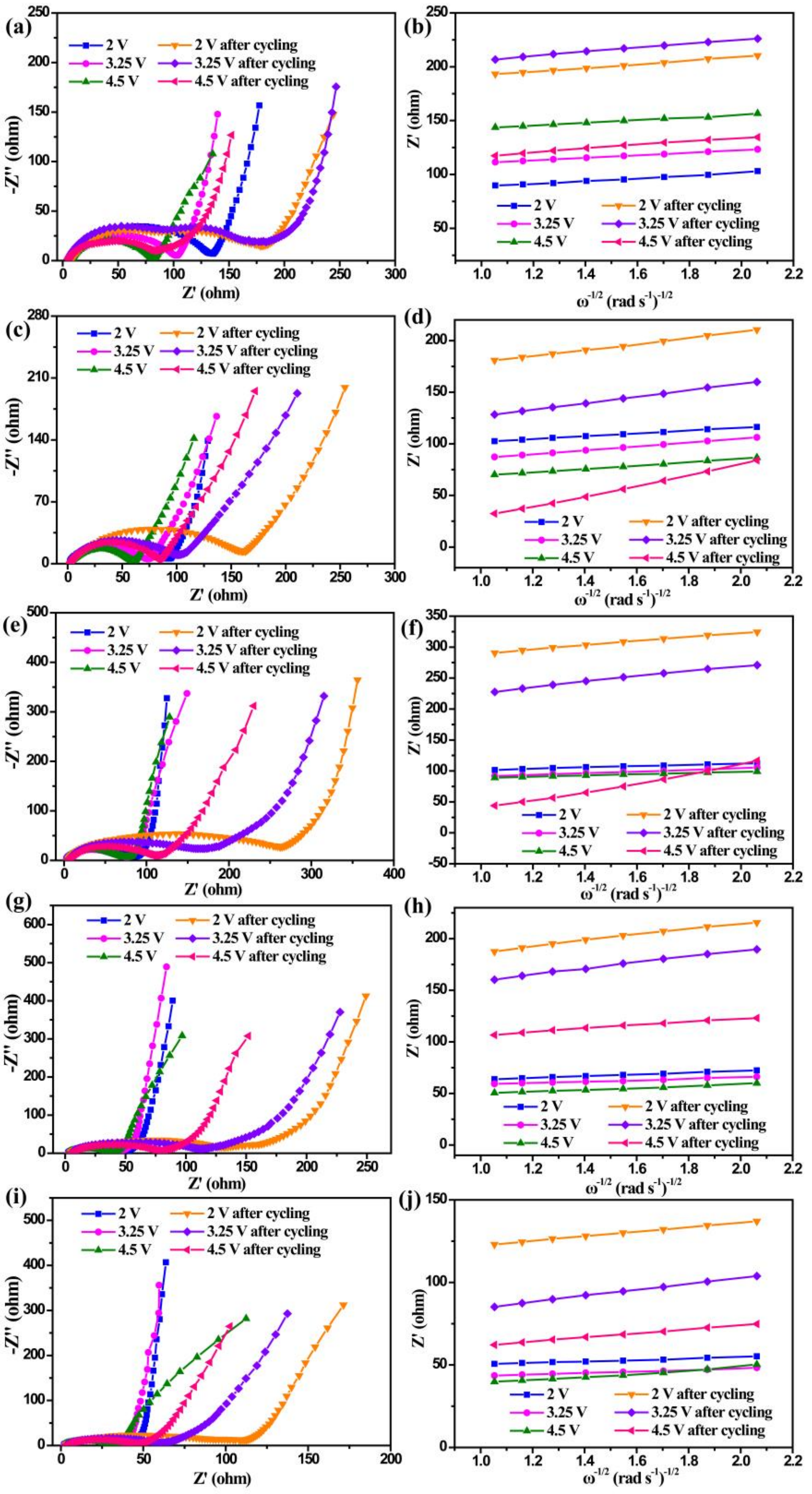

Fig. S8 Nyquist plots and relationships of Z' and $\omega^{-1/2}$ in the low frequency region of (**a**, **b**) Zn_{100}-APC, (**c**, **d**) $Zn_{75}Co_{25}$-APC, (**e**, **f**) $Zn_{50}Co_{50}$-APC, (**g**, **h**) $Zn_{25}Co_{75}$-APC, (**i**, **j**) Co_{100}-APC

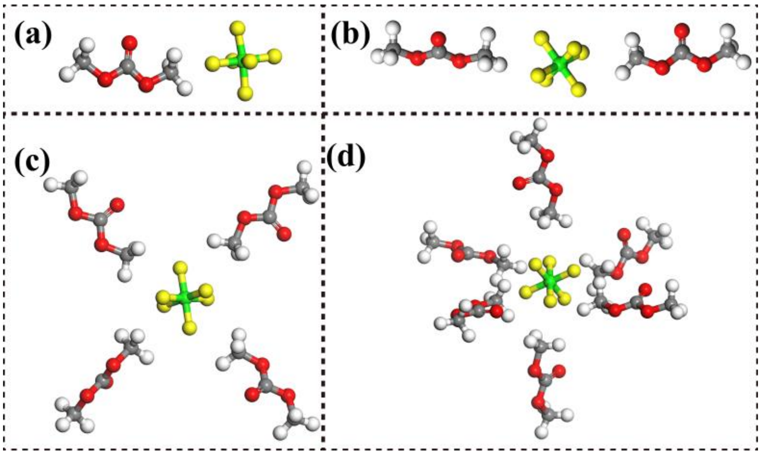

Fig. S9 Optimized solvation structures of $PF_6^-(DMC)_i$ by DFT calculations: (**a**) $PF_6^-(DMC)_1$, (b) $PF_6^-(DMC)_2$, (c) $PF_6^-(DMC)_4$ and (**d**) $PF_6^-(DMC)_6$

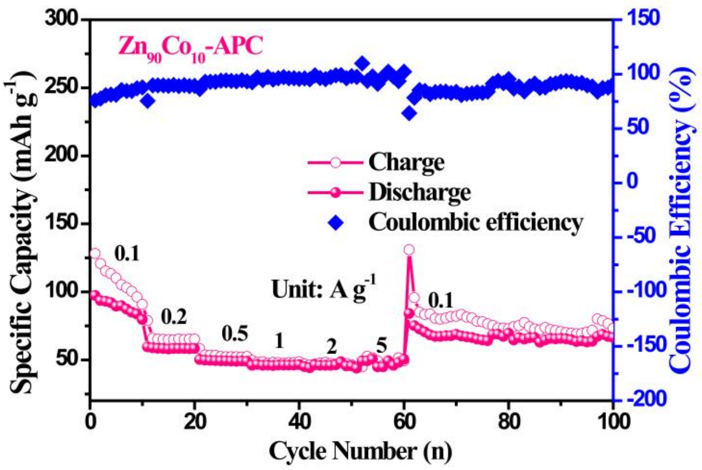

Fig. S10 The coulombic efficiency of the $Zn_{90}Co_{10}$ –APC

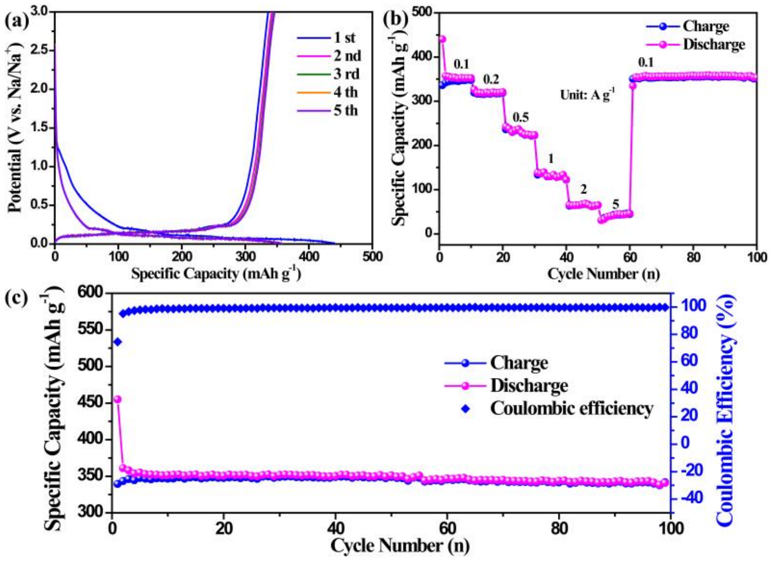

Fig. S11 (**a**) GCD profiles at 0.1 A g^{-1}, (**b**) Rate capability at different current densities and (**c**) Cycling performance at 0.1 A g^{-1} of commercialize graphite anode

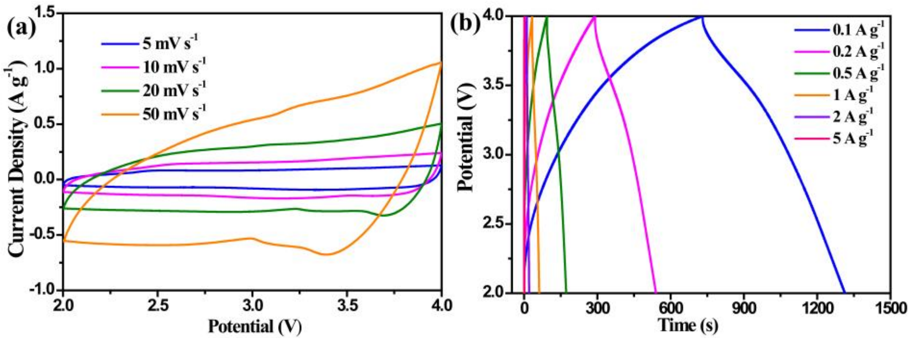

Fig. S12 (**a**) CV curves and (**b**) GCD profiles of PLG//AC LIC

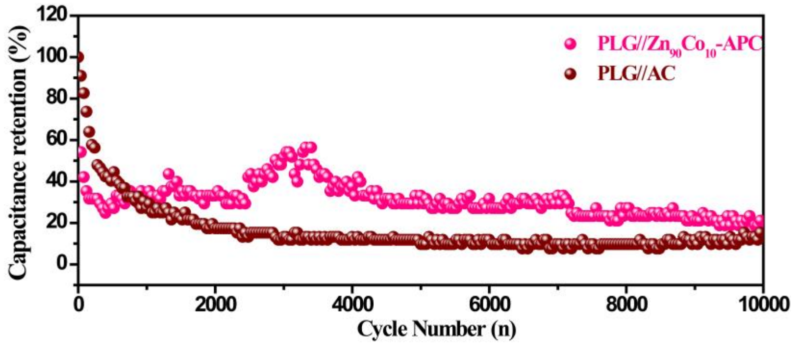

Fig. S13 Cycling stability of PLG//$Zn_{90}Co_{10}$-APC and PLG//AC LICs at 1 A g^{-1} for 10000 cycles within 2-4.0 V.

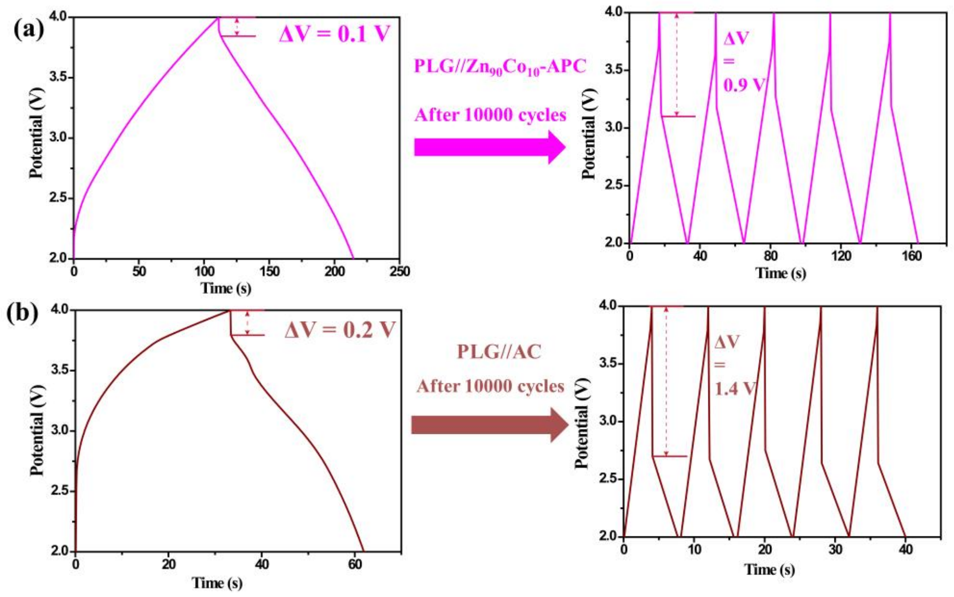

Fig. S14 Comparison of potential drops for PLG//$Zn_{90}Co_{10}$-APC and PLG//AC LICs during the cyclic process

Table S1 XPS elemental contents of Zn_xCo_{100-x}-PCs and Zn_xCo_{100-x}-APCs

Sample	C (at%)	N (at%)	O (at%)
Zn_{100}-PC	67.93	20.59	11.48
$Zn_{75}Co_{25}$-PC	70.94	19.43	9.62
$Zn_{50}Co_{50}$-PC	64.99	9.95	25.06
$Zn_{25}Co_{75}$-PC	75.04	8.69	16.27
Co_{100}-PC	86.55	6.29	7.16
Zn_{100}-APC	81.49	7.47	10.59
$Zn_{75}Co_{25}$-APC	84.28	6.34	9.38
$Zn_{50}Co_{50}$-APC	87.32	5.85	6.83
$Zn_{25}Co_{75}$-APC	89.22	4.64	6.14
Co_{100}-APC	90.60	3.19	6.21

Table S2 The specific calculated values of solvation energies of $PF_6^-(EC)_i$ (i =1, 2, 4, 6) structures

i	ΔE_{solv} (kcal mol^{-1})
1	-15.4672
2	-26.9473
4	-55.0242
6	-71.8422

Table S3 The specific calculated values of solvation energies of $PF_6^-(DMC)_i$ (i =1, 2, 4, 6) structures

i	ΔE_{solv} (kcal mol^{-1})
1	-6.7610
2	-12.9980
4	-26.1158
6	-39.4399
